# A Novel Role for Corneal Pachymetry in Planning Cataract Surgery by Determining Changes in Spherical Equivalent Resulting from a Previous LASIK Treatment

**DOI:** 10.1155/2023/2261831

**Published:** 2023-05-31

**Authors:** Arthur Hammer, Tjebo F. C. Heeren, Romesh Angunawela, John Marshall, Kamran Saha

**Affiliations:** ^1^Moorfields Eye Hospital NHS Foundation Trust, London, UK; ^2^OCL Vision, London, UK; ^3^University College London, Institute of Ophthalmology, London, UK

## Abstract

**Objectives:**

To provide a metric to differentiate between hyperopic and myopic ablation of a prior LASIK treatment based on the corneal pachymetry profile after laser vision correction (LVC).

**Methods:**

Pachymetry data were retrospectively recovered from patients who had previous LASIK for refractive purposes between 2019 and 2020. Patients with any corneal disorder were excluded. Ablation spherical equivalent was predicted from the central to semiperipheral corneal thickness (CPT) ratio, both values were provided by using the Pentacam user interface software (UI), and values were computed from extracted raw pachymetry data.

**Results:**

Data of 157 eyes of 81 patients were collected, of which data were analysed for 73 eyes of 73 patients to avoid concurrence of measurements in both eyes per subject (42% female; mean age 40.9; SD 12.8). The CPT ratio cutoff for distinction between myopic and hyperopic LASIK was 0.86 for Pentacam UI data. Sensitivity and specificity were 0.7 and 0.95, respectively. Accuracy increased with computation of the CPT ratio based on extracted raw data with sensitivity and specificity of 0.87 and 0.99, respectively. There was a marked linear correlation between the CPT ratio and the ablation spherical equivalent (*R*^2^ = 0.93).

**Conclusions:**

CPT ratio cutoffs can correctly classify if a cornea previously had a hyperopic versus myopic LASIK surgery and estimate the ablation spherical equivalent of such treatment. This could prove useful for increased accuracy of intraocular lens (IOL) calculations for patients with no historical data of their prior LVC surgery at the time of cataract surgery planning.

## 1. Introduction

Intraocular lens (IOL) power calculations are a central element to today's cataract surgery and accuracy to predict postoperative refraction increases with technological advances. Today's most used formulas are vergence formulas using biometric measurements such as keratometry readings and axial length to estimate the effective lens position [1]. Newer formulas based on ray tracing [2] and artificial intelligence [3–7] are gaining in popularity but do not represent mainstream usage among cataract surgeons [8]. The refractive power of the cornea can be calculated using keratometry measurements, which are based on an estimated constant ratio between the anterior and posterior corneal curvature [9].

After laser vision correction (LVC), the relationship between the anterior and posterior corneal curvature is modified and the artificially assigned value of the keratometric index of the cornea changes accordingly [9]. IOL calculation formulas that do not account for this change result in higher refractive errors after cataract surgery, in particular, a hyperopic shift after myopic excimer laser treatments and to a lesser extent a myopic shift after hyperopic excimer laser treatments [10–12]. Other sources of refractive surprise can be related to anterior corneal curvature measurements outside of the treatment zone and wrong prediction of the effective lens position based on anterior corneal curvature measurements [9].

New formulas have been developed to address this issue. Some of those formulas, such as Haigis L and Barrett true K, can be used without LVC historical data such as the pre-LVC refraction. Average values and regression analysis based on LVC cohort's data can be used in such instances [9]. However, those formulas still require the surgeon to input the type of LVC correction received: hyperopic versus myopic.

With the ever-increasing popularity of LVC surgery, the number of patients seeking cataract surgery who have had prior treatments such as LASIK, PRK, or SMILE is rising. However, in practice, a significant proportion of those patients do not have access to their historical refractive data or to their surgical records.

For this reason, IOL calculation formulas not using refractive historical data, such as Haigis L and Barrett true K (no history) are of paramount interest, but it has been shown that providing patients' refractive historical data improves the accuracy of such formulas [13]. Therefore, in addition to being able to estimate historical data such as the type of LVC correction (myopic versus hyperopic), estimating also the ablation spherical equivalent, in dioptres, applied to a cornea could prove useful to increase the accuracy of those formulas.

It has been shown that the corneal thickness profile from the central to the peripheral cornea of myopic patients from low myopia (<6D) to high myopia (>12D) does not show significant differences [14]. Central corneal thickness (CCT) variation in the population has been extensively studied and yielded controversial results as to the presence of a correlation between the refractive error and CCT [15].

Laser ablation profiles for myopic and hyperopic correction theoretically change the thickness profile of the cornea in an opposite fashion, with myopic treatments thinning the central cornea and hyperopic treatments thinning the semiperipheral cornea [16]. As a consequence, the ratio between the central and semiperipheral corneal thickness should represent the amount of ablation spherical equivalent (SE) related to tissue ablation. Epithelial fluctuations will be discussed later.

This is a pilot study investigating if this central to semiperipheral corneal thickness ratio (CPT ratio) would be suitable as a measure to predict if a cornea previously received a hyperopic versus myopic LASIK treatment and if this CPT ratio could estimate the ablation spherical equivalent of this LASIK treatment.

## 2. Methods

Retrospective pachymetric data were extracted from a corneal tomographer (Pentacam, OCULUS Optikgeräte GmbH, Münchholzhäuser Straße 29, 35582 Wetzlar, Germany) for a pool of patients treated by LASIK for refractive purposes over a 1-year period from 2019 to 2020. No patients with pure astigmatism were included in this study. For most of the patients in this study, the ablation sphere had a higher weight on the ablation spherical equivalent than the ablation cylinder, except for a few hyperopic patients for whom the ablation spherical equivalent was 0 dioptre, in which case the weight of the sphere and cylinder on the ablation spherical equivalent was equal. Pentacam images were acquired by skilled technicians. All procedures have been performed in the same surgical centre (OCL Vision, London, UK) using the same excimer laser platform (Schwind Amaris 1050 RS, SCHWIND eye-tech-solutions GmbH, Mainparkstraße), with a 6 to 7 mm optical zone, and the same wavefront optimised profile. Only patients without preexisting ocular pathology other than refractive errors were included. Patients with corneal scarring, corneal ectasia, corneal dystrophy, or any disorder affecting corneal homeostasis were excluded. Only corneas with one excimer laser treatment were included. No patients were using contact lenses after LVC. Pentacam images acquired at least 6 months after LASIK were used to avoid wound healing bias effects on the measurements.

The main research question was as follows: can a cutoff value of the CPT ratio alone determine if a cornea had a prior hyperopic or myopic treatment, defined as the ablation spherical equivalent of in dioptres? Hyperopic treatment was defined as 0 or more dioptres (D) of ablation spherical equivalent. 0 dioptre of ablation spherical equivalent has been attributed to the hyperopic group because we used the negative cylinder astigmatism notation. Therefore, only hyperopic treatments can lead to an ablation spherical equivalent of 0 dioptre. Receiver-operating characteristics (ROC) curves, specificity, sensitivity, and accuracy were calculated to assess the predictability of the model.

The formula used to calculate the CPT ratio was

(1) $$\begin{array}{c} CPT\;ratio=\frac{central\;corneal\;thickness}{semiperipheral\;corneal\;thickness\;at\;a\;given\;radius}\text{.} \end{array}$$

All data handling and statistical analysis were performed using statistical programming language R [17].

**Figure 1:**
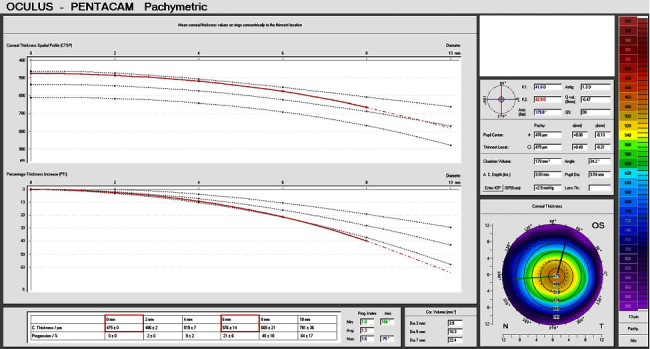
Pachymetry page of the Pentacam tomographer user interface (UI) software. The table at the bottom of the page shows the average pachymetry at the center of the cornea and at different diameters in the periphery. The values shown in red are used to calculate the CPT ratio in this study.

Data were extracted in two ways for separate analysis. First, the CPT ratio was calculated from the corneal thickness data taken from the Pentacam software user interface (Pentacam UI) at a radius of 0 and 3 mm (6 mm diameter), as shown in Figure 1 (Pentacam UI CPT ratio). Second, raw pachymetry data were extracted from the Pentacam using the manufacturer's csv file export function (raw data CPT ratio). Pachymetry measurements are stored in a grid-like fashion, for one measurement every 0.1 mm, from −7 mm to +7 mm in both the *x* and *y* directions. The correct coordinates for extracting the corneal thicknesses at specific radii and angles were obtained by means of simple trigonometry. The average corneal thickness was calculated for every five degrees from 0 to 360 degrees for radii ranging from 1 to 5 mm, in steps of 0.5 mm. Larger radii were not considered useful because measurements regularly had large areas of missing data (pachymetric data rarely reached the 14 mm diameter generously given by the Pentacam software). To explore which radius was the most useful to predict the ablation spherical equivalent from the CPT ratio, we fitted simple linear regression models to the data. As there was concurrence of measurements in both eyes per subject, we decided to only use one eye per patient, which was selected by random sampling. This was performed with the sample function using a seed for reproducibility. The best model performance was evaluated based on R-squared values, as well as on their root mean squared error and the Bayesian information criterion (BIC).

The same one-eyed data were randomly split into a training set (2/3 of the data) and a validation set (1/3 of the data). Receiver-operating characteristics (ROC) were fitted to the training data using the ROCR package [18], whereby “hyperopia” (ablation spherical equivalent greater than and equal to 0 dioptre) was defined as the positive characteristic. Accuracy was estimated as (true positive + true negative)/(predicted positive + predicted negative). Sensitivity was estimated as true positive/positive, and specificity was estimated as true negative/negative. Predictions were then made on the test data. This was repeated 30 times to obtain error estimations.

In a secondary research question, we assessed the performance of the prediction of the ablation spherical equivalent from the CPT ratio based on simple linear models developed from the one-eyed data. Visual exploration showed a marked linear correlation. Therefore, linear regression seemed a viable approach. The Breusch–Pagan test on the data residuals did not reveal significant heteroscedasticity (*p* value =0.63).

As the optical zone or ablation zone might influence the significance of the CPT ratio as a predictive factor, we also tested its significance as a predictive variable by including it as an independent measure in our model.

## 3. Results

Data were collected for 157 eyes of 81 patients. After the exclusion of the eyes that had another laser refractive surgery than LASIK (8 eyes), or if the Pentacam examination was performed less than six months after the LVC treatment (8 eyes), or if there was more than one laser treatment performed for the same eye (1 eye), 140 eyes of 73 patients remained and were used for random sampling of one eye per patient; therefore, 73 eyes of 73 patients remained for analysis (42% female; mean age 40.9; SD 12.8). The mean time of the Pentacam examination after laser treatment was one year (SD 0.2 years).

For the Pentacam UI CPT ratio (Figure 1), the mean cutoff value for the best distinction of previous hyperopic versus myopic ablation was 0.86 (SD 0.01). The mean accuracy was 0.89 (SD 0.06), the mean sensitivity was 0.7 (SD 0.22), and the mean specificity was 0.95 (SD 0.06). Sensitivity and specificity curves for each iteration are shown in Supplemental Figure 1.

Supplemental Figure 2 shows the linear regression for the raw data CPT ratio at different radii of semiperipheral corneal thickness, and the best radius for prediction was 2.5 mm. The mean cutoff value for the best distinction was 0.92 (SD 0.01). The mean accuracy was 0.96 (SD 0.04), the mean sensitivity was 0.87 (SD 0.17), and the mean specificity was 0.99 (SD 0.02).

**Figure 2:**
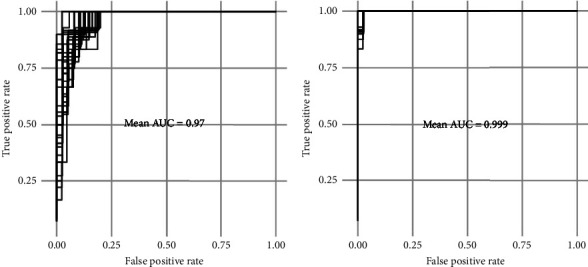
Receiver-operating characteristics (ROC) curves, based on precomputed values provided by Pentacam UI software output (a) and on extracted raw pachymetry data (b). Training was repeated on 2/3 of 30 independent random samples of the one-eyed data (one random eye for each participant). AUC: area under the curve.

Figure 2 shows the area under the curve (AUC), a measure of the accuracy of the prediction (1 being ideal). AUC was 0.97 for the Pentacam UI CPT ratio (Figure 2(a)) and 0.999 for the raw data CPT ratio (Figure 2(b)).

**Figure 3:**
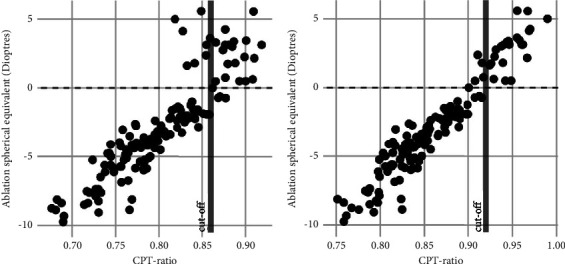
Spherical equivalent of LASIK treatments as a function of the postoperative central to semiperipheral corneal thickness (CPT) ratio. (a) The CPT ratio calculated from the precomputed values of central and semiperipheral (at 3 mm radius) corneal thicknesses provided by the Pentacam UI software and (b) the CPT ratio calculated from the extracted raw data values of central and semiperipheral (at 2.5 mm radius) corneal thicknesses provided by the .csv export function of the Pentacam software. The computed mean cutoff is shown for both panels. Using raw data increased accuracy substantially (b) with dots in the extremes (lower left and upper right quadrants) showing less spread from the ideal linear regression.

Figure 3 shows the linear regression between CPT ratio and ablation spherical equivalent. The variance of the residuals is slightly higher toward hyperopic values when using the Pentacam UI CPT ratio, showing a tendency of outliers in the hyperopic range with a CPT ratio indicating a myopic ablation (Figure 3(a)). However, when using the raw data CPT ratio, the variance is more uniformly distributed and smaller throughout the entire range, and almost no outliers can be seen. As such, the magnitude of the error is smaller (Figure 3(b)). The formula obtained from the linear regression model for the Pentacam UI CPT ratio (Figures 1 and 3(a)) was

(2) $$\begin{array}{c} ablation\;spherical\;equivalent\;\left(in\;dioptres\right)=-49.14+57.62\;x\;CPT\;ratio\text{.} \end{array}$$

Table 1 shows the ablation spherical equivalent predicted from the Pentacam UI CPT ratio values comprised between 0.67 and 0.95 as per the previous equation.

The Pentacam UI CPT ratio is a good measure to predict the ablation spherical equivalent (*R*^2^ = 0.83; *p* value <0.001) (Figure 3(a)). The prediction performance increased considerably when using the manually computed raw data CPT ratio at 2.5 mm radius (*R*^2^ = 0.93; *p* value <0.001) (Figure 3(b)). This means that the raw data CPT ratio at 2.5 mm radius explained 93% of the variance of the ablation spherical equivalent.

The optical zone diameter ranged from 6.0 to 7.0 mm (mean 6.4 mm; SD 0.2 mm), and the ablation zone diameter ranged from 6.8 to 7.8 mm (mean 7.6 mm; SD 0.4 mm). As shown in Supplemental Figure 3, both optical and ablation zones had no clear linear relationship with the ablation spherical equivalent; thus, we decided to not include them as independent variables in our linear model. As the performance of our simple linear model was already adequate, we decided not to fit an exponential model to the data.

## 4. Discussion

Intraocular lens selection is a critical aspect of cataract surgery planning and the most challenging for LVC patients for whom the current formulas still leave a likelihood of refractive surprise. Knowing the pre-LVC refractive status helps the prediction of cataract surgery refractive outcomes for LVC patients [14]. When using IOL calculation formulas such as Haigis-L and Barrett true K (no history), it is required to specify the type of prior LVC treatment (myopic versus hyperopic). In fact, one can observe the significant variations in IOL power suggestions, for the same patient's data, depending on the type of prior LVC treatment chosen at the time of IOL power calculation. To date, without records of the prior refractive status, the treating clinician is limited to subjective and arbitrary evaluation of sagittal and tangential curvature maps which could give clues if there might have been a prior myopic or hyperopic treatment [19]. However, this method of evaluation lacks standardisation and validation.

This pilot study shows that the post-LVC central to semiperipheral corneal thickness ratio (CPT ratio) can accurately predict the type of LASIK ablation profile (hyperopic vs myopic) that was used, as well as provide an estimate of the ablation spherical equivalent in dioptres. This could in fact be predicted from the ablation profiles used by most laser platforms with mostly central ablations for myopic treatments, therefore decreasing the CPT ratio, and inversely for hyperopic treatments consisting of more peripheral ablations, therefore increasing this ratio.

Our study shows that the CPT ratio can serve as a valid cutoff value to distinguish between prior myopic versus hyperopic LVC. This information is required even when using formulas not requiring pre-LVC refractive data such as Haigis-L and Barrett true K (no history). In addition, studies have shown that additional knowledge of pre-LVC refractive status increases the accuracy of IOL calculations [10, 14]. The pre-LVC spherical equivalent can be estimated from the manifest refraction at the time of cataract surgery planning and the estimated ablation spherical equivalent given by the CPT ratio as follows:

(3) $$\begin{array}{c} Estimated\;pre-LVC\;SE\left(D\right)=Manifest\;refraction\;SE\left(D\right)+Ablation\;SE\left(D\right)\;estimated\;from\;the\;CPT\;ratio\text{.} \end{array}$$

The ablation spherical equivalent could be reasonably estimated from the CPT ratio alone. This ratio can be calculated by cataract surgeons using corneal thicknesses displayed on the Pentacam user interface software or the corneal tomographer of their choice. Alternatively, it can be calculated from the raw pachymetry data for improved accuracy. Thus, using the CPT ratio can help clinicians make a decision affecting the refractive outcome of cataract surgery in LVC patients without historical refractive data by increasing the accuracy of biometric predictions.

Hashemi et al. [20] observed in an Iranian population that the corneal thickness decreased with age by 0.65 *μ*m per year peripherally and 0.25 *μ*m per year centrally. As a result, adjusting the CPT ratio for age of the population might help improve the accuracy of the prediction of the ablation spherical equivalent. However, we do not yet fully understand whether similar changes would be expected in ageing eyes after LVC.

Alternatively, IOL calculation methods such as ray tracing do not require historical data and do not depend on keratometry indices. Ray tracing allows the calculation of the real anterior and posterior corneal curvatures and is not associated with measurement radius issues as calculations can be done on any diameter. Also, ray tracing allows corneal aberrations to be included in the IOL calculations [2, 21]. Unfortunately, so far, ray tracing has shown controversial results in the literature and is not yet widely available [22, 23].

Intraoperative aberrometry has been used to improve IOL choice during cataract surgery [9, 24]. It has been shown that for LVC patients without historical data, intraoperative aberrometry using the Optiwave Refractive Analysis (ORA) System (WaveTec Vision Systems Inc, Aliso Viejo, CA) wavefront aberrometer improved the postoperative refractive prediction error. However, this system is also not widely available, is costly and has shown controversial results [25].

One possible constraint of the present study is that all data analysed were recovered from one surgical centre with one excimer laser using the same wavefront-optimised profile for LASIK treatments. Variations are to be expected between our dataset and other surgical centres using different lasers, surgical procedures (PRK, trans-PRK, LASEK, SMILE, etc...) and ablation profiles (conventional, wavefront-optimised, wavefront-guided, topography-guided, Q-based, etc...), and those variables will change with technological advances. Further studies with data from multiple centres using different lasers, treatment modalities and ablation profiles are needed to validate our results in other settings.

In addition, it has been shown previously that the excimer laser stromal ablation and the flap side cut in the case of LASIK both induce biomechanical changes in the cornea inducing refractive changes which are, to date, difficult to predict with a current lack of a comprehensive biomechanical model. When collagen fibres and lamellae are cut, stress redistribution among the uncut fibres induces a hyperopic shift. As such, the stroma thins where the lamellae are cut and the surrounding stroma thickens [26].

It must be noted that, as shown previously, the epithelial thickness distribution changes after LVC. In particular, the epithelium overlying ablation areas thickens proportionally to the ablation depth and the anterior corneal curvature flattening rate. Conversely, the epithelium thins overlying areas of anterior corneal curvature steepening. Thus, epithelial remodelling participates in the regression toward the prior ametropia. [27–29]. Therefore, using a device (e.g. anterior segment OCT, very high-frequency ultrasound) capable of subtracting the epithelial thickness from the total corneal thickness and thus allowing the stromal thickness analysis could improve the CPT ratio's accuracy. As a result, this could improve the accuracy of the distinction between prior hyperopic and myopic LVC and their estimated ablation spherical equivalent.

Furthermore, tomography measurements show interdevice variability which in turn can affect the predictability of the CPT ratio [30]. More studies are needed to establish a reliable CPT ratio using other corneal tomographer devices than the Pentacam. In addition, corneal tomographers also show, to some extent, intradevice measurements variability [31].

As shown in our analysis of the optical/ablation zones, we could not find a significant correlation with the CPT ratio, nor with the ablation spherical equivalent. Also, as previously described in the literature, most treatment planning systems of excimer lasers use optical zones ranging between 6 and 6.5 mm with a general trend toward larger optical zones. Those are comparable to the optical zones used in this study [32–35]. Therefore, the principle of the CPT ratio may be applied to excimer lasers from other manufacturers than the one used in this study.

In conclusion, we have shown that the CPT ratio can differentiate, with reasonable accuracy, between a prior myopic versus hyperopic LASIK treatment and even predict the ablation spherical equivalent of such treatments. Whilst the present study did not include any presbyopic treatment, similar considerations would apply. Incorporation of this metric into current corneal tomographers, anterior segment OCTs, biometers, and other devices allowing analysis of the corneal thickness profile could help inform clinicians regarding prior LVC and be utilised to improve cataract surgery outcomes in LVC patients without historical refractive data or surgical records.

## Figures and Tables

**Table 1:** Ablation spherical equivalent (SE) predicted from the central to semiperipheral corneal thickness ratio (CPT ratio) calculated from the precomputed values obtained from the pentacam user interface software.

CPT ratio	SE (D)
0.67	−10.5
0.69	−9.4
0.71	−8.2
0.73	−7.1
0.75	−5.9
0.77	−4.8
0.79	−3.6
0.81	−2.5
0.83	−1.3
0.85	−0.2
0.87	1.0
0.89	2.1
0.91	3.3
0.93	4.5
0.95	5.6

D = dioptre; CPT = central to semiperipheral; SE = spherical equivalent.

## Data Availability

The data used to support the findings of this study are included within the article.

## Supplementary Materials

Supplementary Materials Supplemental Figure 1: postoperative central to semiperipheral corneal thickness (CPT) ratio, based on precomputed values provided by Pentacam UI software (A) and on extracted pachymetry raw data (B). Training was repeated on 2/3 of 30 independent random samples of the one-eyed data (one random eye for each participant). Supplemental Figure 2: ablation spherical equivalent in dioptres as a function of the postoperative central to semiperipheral corneal thickness (CPT) ratio, shown for different radii from 1 mm to 5 mm (one random eye per patient). CPT ratio was computed based on extracted pachymetry raw data. The best prediction performance was found at 2.5 mm. Supplemental Figure 3: ablation zone and optical zone relation to the ablation spherical equivalent. The ablation zone seemed to show a rather complex relation to the previous treatment (where *y* is not a function of *x*), and the optical zone vs the ablation spherical equivalent showed a rather sinusoidal relation. Thus, in both cases, there was no simple way of including them as variables in our linear model.
