# Supplementary figures and images for: A Novel Role for Corneal Pachymetry in Planning Cataract Surgery by Determining Changes in Spherical Equivalent Resulting from a Previous LASIK Treatment
Source: J Ophthalmol. 2023 May 31;2023:2261831. doi: 10.1155/2023/2261831 (PMC10362985; doi:10.1155/2023/2261831)

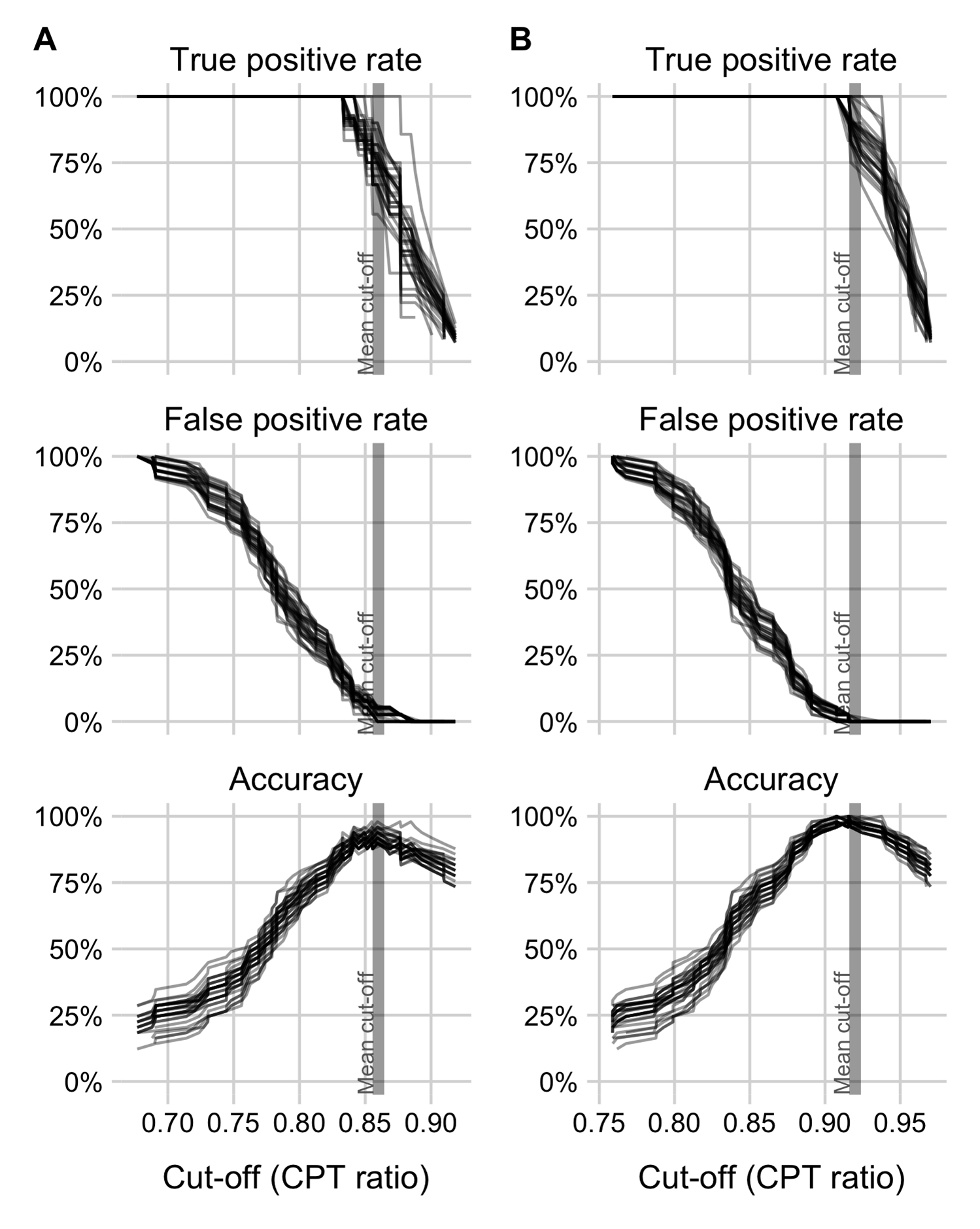

Supplement: Supplementary Materials — Supplemental Figure 1: postoperative central to semiperipheral corneal thickness (CPT) ratio, based on precomputed values provided by Pentacam UI software (A) and on extracted pachymetry raw data (B). Training was repeated on 2/3 of 30 independent random samples of the one-eyed data (one random eye for each participant). Supplemental Figure 2: ablation spherical equivalent in dioptres as a function of the postoperative central to semiperipheral corneal thickness (CPT) ratio, shown for different radii from 1 mm to 5 mm (one random eye per patient). CPT ratio was computed based on extracted pachymetry raw data. The best prediction performance was found at 2.5 mm. Supplemental Figure 3: ablation zone and optical zone relation to the ablation spherical equivalent. The ablation zone seemed to show a rather complex relation to the previous treatment (where y is not a function of x), and the optical zone vs the ablation spherical equivalent showed a rather sinusoidal relation. Thus, in both cases, there was no simple way of including them as variables in our linear model. [file 2261831.f1.zip › supp_fig_1 copy.jpg]

**Ablation spherical equivalent (Dioptres)**

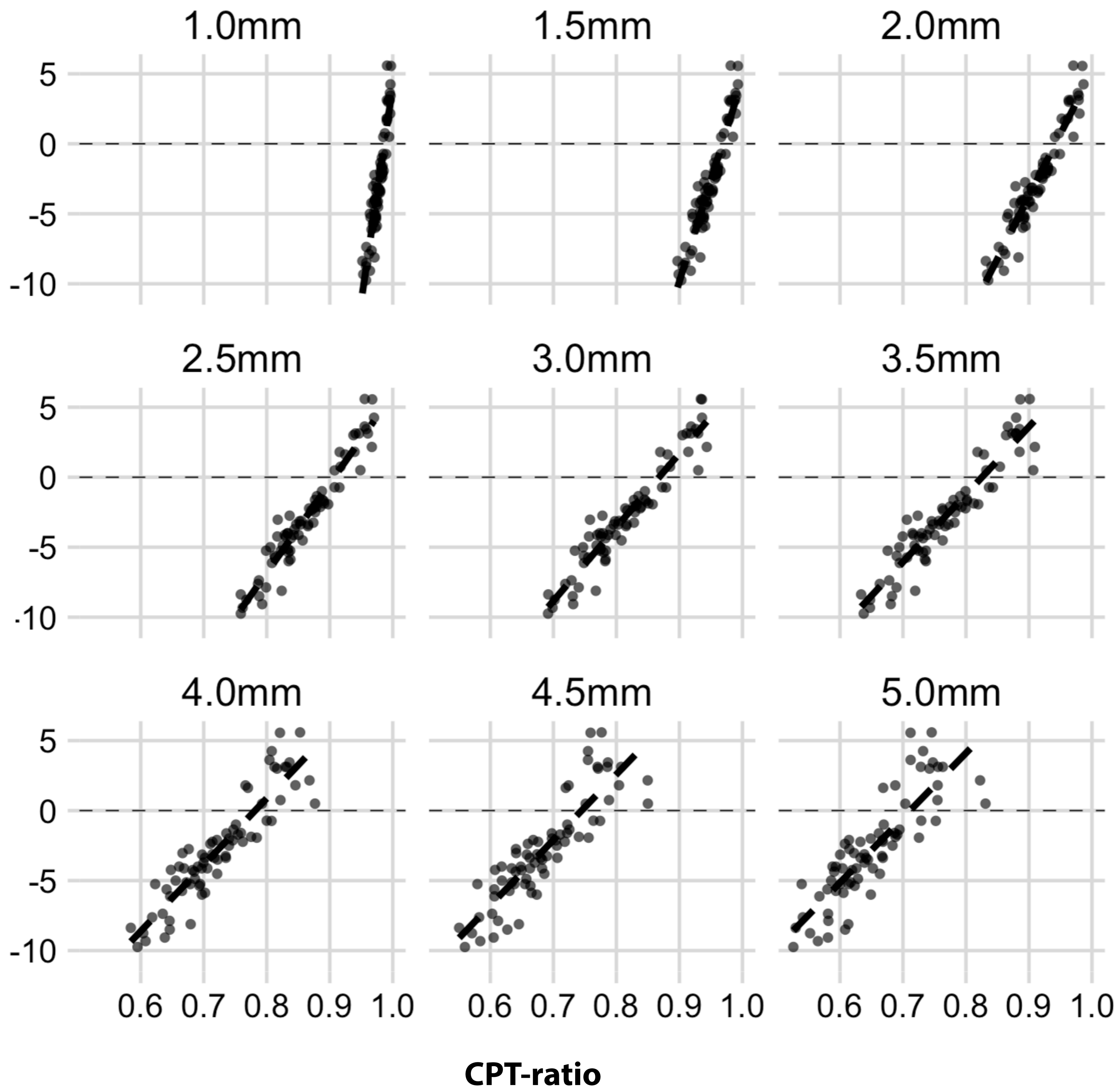

Supplement: Supplementary Materials — Supplemental Figure 1: postoperative central to semiperipheral corneal thickness (CPT) ratio, based on precomputed values provided by Pentacam UI software (A) and on extracted pachymetry raw data (B). Training was repeated on 2/3 of 30 independent random samples of the one-eyed data (one random eye for each participant). Supplemental Figure 2: ablation spherical equivalent in dioptres as a function of the postoperative central to semiperipheral corneal thickness (CPT) ratio, shown for different radii from 1 mm to 5 mm (one random eye per patient). CPT ratio was computed based on extracted pachymetry raw data. The best prediction performance was found at 2.5 mm. Supplemental Figure 3: ablation zone and optical zone relation to the ablation spherical equivalent. The ablation zone seemed to show a rather complex relation to the previous treatment (where y is not a function of x), and the optical zone vs the ablation spherical equivalent showed a rather sinusoidal relation. Thus, in both cases, there was no simple way of including them as variables in our linear model. [file 2261831.f1.zip › sup fig 2.pdf]

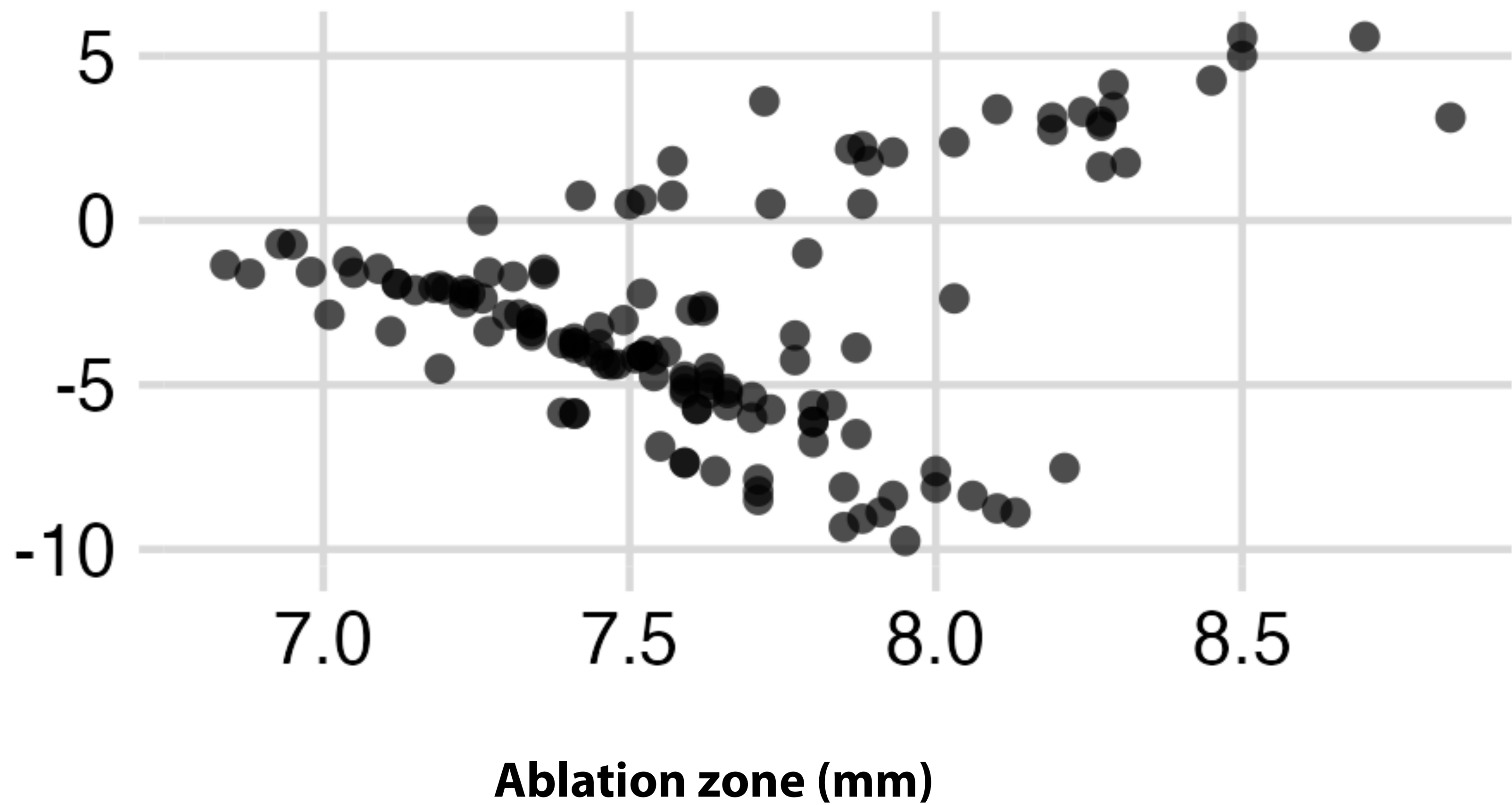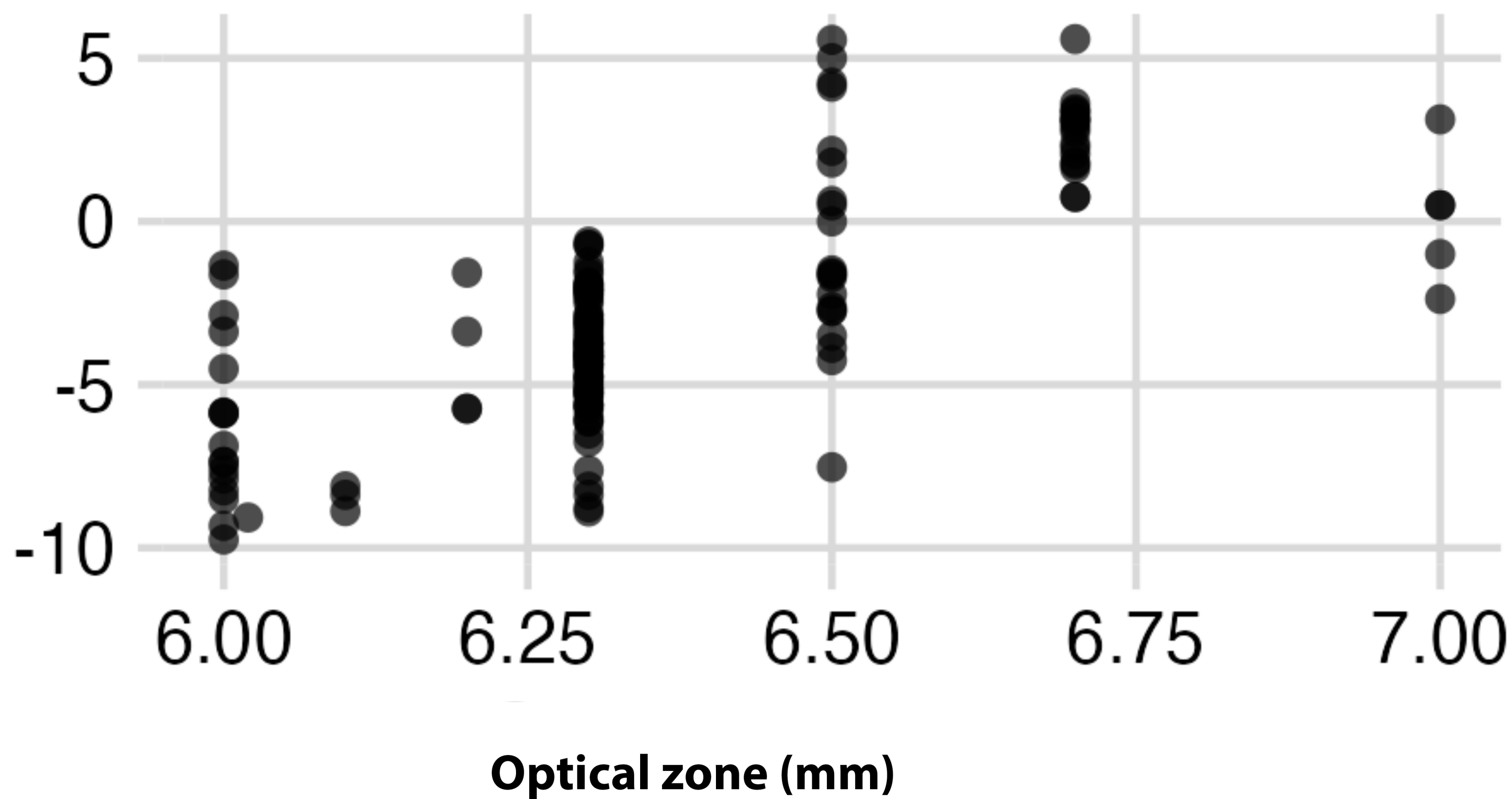

Supplement: Supplementary Materials — Supplemental Figure 1: postoperative central to semiperipheral corneal thickness (CPT) ratio, based on precomputed values provided by Pentacam UI software (A) and on extracted pachymetry raw data (B). Training was repeated on 2/3 of 30 independent random samples of the one-eyed data (one random eye for each participant). Supplemental Figure 2: ablation spherical equivalent in dioptres as a function of the postoperative central to semiperipheral corneal thickness (CPT) ratio, shown for different radii from 1 mm to 5 mm (one random eye per patient). CPT ratio was computed based on extracted pachymetry raw data. The best prediction performance was found at 2.5 mm. Supplemental Figure 3: ablation zone and optical zone relation to the ablation spherical equivalent. The ablation zone seemed to show a rather complex relation to the previous treatment (where y is not a function of x), and the optical zone vs the ablation spherical equivalent showed a rather sinusoidal relation. Thus, in both cases, there was no simple way of including them as variables in our linear model. [file 2261831.f1.zip › Sup fig 3.pdf]
